# Investigating the effects of Functional Ice (FICE) on *Salmonella*-food safety, microbial spoilage and quality of raw poultry thigh meat during refrigerated storage

**DOI:** 10.1371/journal.pone.0234781

**Published:** 2020-06-19

**Authors:** Jasmine Kataria, Laura J. Garner, Emefa A. Monu, Yifen Wang, Amit Morey

**Affiliations:** 1 Department of Poultry Science, Auburn University, Auburn, Alabama, United States of America; 2 Department of Biosystems Engineering, Auburn University, Auburn, Alabama, United States of America; University of Campinas, BRAZIL

## Abstract

In meat processing, antimicrobial treatment applied during slaughter and deboning may not control pathogens and spoilage organisms during subsequent transportation and storage. “Functional Ice” (FICE), an innovation over traditional ice, was investigated for its effects on food safety, shelf life, and quality of raw poultry thigh meat during refrigerated storage. FICE was prepared by freezing aqueous solutions of sodium tripolyphosphate (STPP) (2.5% and 5% w/v) and sodium lactate-sodium diacetate (SL-SD) (1% and 2.5% v/v). Potable water was used to prepare ice for the control treatment. Thigh meat inoculated with *Salmonella* Typhimurium (10^8^ CFU/sample) was placed in FICE treatments, stored at 4 °C and sampled at 0, 12, 24, 36 and 48 h (n = 375). Weight pick-up was recorded for the uninoculated thighs. Additionally, shelf life and quality were evaluated for 8 days on tray-packed thighs that were stored in FICE treatments for 48 h (STPP 5%, and SL-SD 2.5%). Differences among treatments were determined using ANOVA with LSMeans (p ≤ 0.05). Results indicated that inoculated thighs stored in individual STPP 5%, and SL-SD 2.5% treatments lead to a significant reduction in *Salmonella* Typhimurium compared to the control (p ≤ 0.05) after 48 h of storage. FICE treated thighs showed higher yields, lower cook loss, and an extended shelf life of 1–2 days, without any color changes. FICE has the potential to improve food safety and shelf life while improving the yields and quality during storage and transportation of raw poultry meat.

## Introduction

*Salmonella* is a major foodborne pathogen commonly associated with raw poultry and poultry products causing 1.2 million illnesses, 23,000 hospitalizations and 450 deaths, annually in the United States [1]. Between 2009 and 2015, 15% of the top 5 pathogen-food category pair outbreaks were attributed to Salmonellosis associated with chicken consumption [2]. To reduce the prevalence of *Salmonella*, poultry processors apply antimicrobial interventions at various steps of poultry processing [3]. Currently, refrigeration is the most common intervention during storage and transportation to improve food safety. *Salmonella* can survive at refrigeration temperature on raw chicken meat [4,5] and further temperature abuse can initiate pathogen growth leading to food safety concerns [6]. Additionally, psychrotrophic microorganisms can grow and spoil meat under refrigerated storage and transportation leading to food waste [7, 3]. There is a need to develop intervention strategies to improve food safety and reduce spoilage during storage and transportation.

Ice is commonly used during the bulk storage and transportation of foods as it reduces the product temperature due to its high cooling capacity, resulting in a reduced rate of microbiological and biochemical degradation [8]. In addition to ice, commercial poultry processors use dry ice for the packaging and shipment of raw poultry meat [9]. Dry ice provides a greater cooling effect (-78.5 °C) than the regular ice (0–1 °C), thereby exhibiting a bacteriostatic effect on the microorganisms on the fresh meat [10]. Fratamico et al. developed another antimicrobial intervention, ALIGAL Blue Ice (ABI) for the packaging and transportation of food. ABI is an ozonated dry ice which incorporates both the enhanced cooling capacity of dry ice and antimicrobial action of ozone to suppress the growth of pathogens and improve shelf life [9]. However, dry ice, either used alone or in combination with ozone, has some potential hazards such as chances of explosion, suffocation, and contact hazards during transportation [11]. It requires great handling skills, which might limit its use. In addition, dry ice does not facilitate the improvement of meat quality parameters that are important to processors and consumers, such as yield of raw meat, color, texture, and cooking yields during the storage and transportation.

Another intervention, peracetic acid ice also known as frozen biocidal-active ice was invented to be used for the storage and transportation of poultry products commercially to inhibit the growth of spoilage and pathogenic microorganisms [12]. Despite, the reported antimicrobial effect of peracetic acid, several occupational hazards have been reported due to the high reactivity of peracetic acid [13]. There is limited published literature on the effect of peracetic acid ice on the microbial growth and quality characteristics of poultry meat during storage and transportation which restricts its application on a commercial scale.

There is a need to develop of a simple, feasible and safe antimicrobial ice which could effectively control the spoilage and pathogenic organisms while preserving the quality aspects of poultry meat during storage and transportation. An innovative product known as “Functional Ice” (FICE) was developed by freezing food-grade ingredient solutions. Functional ice acts as a “Sustained Release Mechanism” for the ingredients as FICE melts, it would ideally serve the following functions: (i) improve food safety by actively eliminating foodborne pathogens; (ii) increase shelf life by actively suppressing spoilage microorganisms; (iii) provide lower cooling temperatures thus improving food safety and reducing spoilage risks due to temperature abuse during storage and transportation; and (iv) maintain quality and yield as they are highly important for the processors and consumers.

The ingredients selected for the study were sodium tripolyphosphate (STPP) and sodium lactate + diacetate (SL-SD) based on their multi-functional properties of inhibiting growth of microorganisms, meanwhile, maintaining meat quality and yield. These two ingredients are generally recognized as safe (GRAS) and are commonly used in meat and poultry products [14]. Food grade sodium tripolyphosphate functions as follows: (i) exhibits antimicrobial activity; (ii) increase the water holding capacity of meat; (iii) reduces the cook loss %; (iv) increases the yield; and (v) improve the textural properties of meat [15, 16]. The antibacterial mechanism of STPP has been reported due to the sequestration of metallic ions in the cell wall resulting in the loss of cell wall integrity, thereby inhibiting the growth of microorganisms [17, 18]. The organic acid salts, sodium lactate, and diacetate are believed to delay the growth of microorganisms by extending the lag phase [19, 20]. Besides, sodium lactate and sodium diacetate have been reported to give higher cooking yields and improved textural properties of meat [21, 22]. These ingredients have demonstrated antimicrobial and other functional properties such as antioxidant activity, chelation, and preservative properties in food, but there is limited research on their use in the form of ice in the poultry meat [23].

Therefore, the objective of the current research was to evaluate the effects of different FICE formulations on the survival of *Salmonella* Typhimurium inoculated on poultry meat and to investigate its effects on the yield, shelf life, and quality parameters of raw poultry meat under refrigerated storage.

## Material and methods

### Preparation of FICE

All the FICE treatments were prepared in the research kitchen at the Department of Poultry Science, Auburn University. The four FICE treatments were (i) STPP 2.5% (w/v) (Brifisol^®^, ICL Food Specialties, St. Louis, MO, U.S.A. (ii) STPP 5% (w/v) (Brifisol) (iii) Sodium lactate and sodium diacetate (SL-SD) 1% (v/v) (Opti. SD4 (SL 56% and SD 4%), Corbion Purac, NE, U.S.A.) (iv) Sodium lactate and sodium diacetate (SL-SD) 2.5% (v/v) (Corbion). The SL-SD 1% treatment contained the final SL and SD concentration of 0.56% and 0.04%, respectively while SL-SD 2.5% treatment had 1.4% and 0.1%, respectively. Ice made from potable water from the public supply in the University served as a control treatment. FICE solutions were prepared by completely dissolving the ingredients in individual containers of potable water using a hand blender (Bella Immersion Blender, #HB1908KB-ET). The pH of all the treatments was recorded using a pH meter (Hach, Model No. H170G, Loveland, CO, U.S.A.). The pH of the solutions was 9.04, 9.11, 6.0, 5.94 and 6.04 for the STPP 2.5%, STPP 5%, SL-SD 1%, SL-SD 2.5% treatments and control, respectively. FICE solutions were poured into the ice cube trays (Sterilite, 29.97 × 12.06 × 4.32 cm) and frozen in a walk-in freezer (-20 °C) for 24–48 h. Frozen FICE cubes (4 × 2.8 × 3 cm approx.) of each treatment were taken out of the ice trays and stored in the individual plastic bags in the freezer (-20 °C) until further use.

### Effect of FICE on the survival of *Salmonella* Typhimurium inoculated on thigh meat

#### Preparation of *Salmonella* inoculum

A nalidixic acid resistant marker strain of *Salmonella* Typhimurium (isolated from the Auburn University Poultry Research Farm and selected for resistance to nalidixic acid) was cultured in Brain-Heart Infusion broth (BHI; Acumedia Manufacturers, Lansing, MI, U.S.A.) for 24 h at 37 °C. A nalidixic acid resistant strain was chosen to inoculate the meat to minimize potential interference with the natural *Salmonella* on the meat and to accurately quantify the effects of the treatments on the pathogen reduction. Further, a loopful of culture was streaked on Xylose-Lysine-Tergitol 4 agar (XLT4; Acumedia Manufacturers, Lansing, MI, U.S.A.) containing 35 μL/mL of nalidixic acid (Sigma-Aldrich, St. Louis, MO, U.S.A.) and incubated at 37 °C for 24 h. Typical, isolated *S*. Typhimurium colonies were inoculated in fresh BHI broth (with nalidixic acid 35 μL/mL) and incubated for 20–24 h at 37 °C. One mL of the *Salmonella* culture was sub-inoculated in 99 mL BHI media flasks and incubated for 12 h. After 12 h, the cultures (early-stationary phase) were centrifuged (Sorvall Legent RT+ Centrifuge, Thermo Scientific, Thermo Electron Corp., Osterode am Harz, Germany) at 8000 × g for 10 min, the supernatant was decanted and the pellet was resuspended with 1% phosphate buffered saline (PBS; Fisher Scientific, Fair Lawn, NJ, U.S.A.). The centrifugation steps were repeated two times and final pellet was resuspended in PBS to obtain a stock culture of 10^9^ CFU/mL *Salmonella* Typhimurium. *Salmonella* concentration in the stock culture was confirmed by direct plating on XLT4 containing 35 μL/mL of nalidixic acid [24, 25].

#### Inoculation of thighs with *Salmonella*

Fresh, boneless, skinless, raw, chicken thighs (average weight 0.21 kg) were obtained from a local commercial poultry processing facility and transported to Auburn University (~65 km) within 1 h of processing and maintained at 4 °C for approximately 2–3 h prior to inoculation. For each replication, individual thighs were inoculated with 100 μL of the stock culture of *Salmonella* Typhimurium (10^8^ CFU/sample). The inoculum was evenly spread on the surface of thighs with a sterile spreader and placed in sterile aluminum pans, covered and placed in the refrigerator (4 °C for 60 min) to allow bacterial attachment on the surface of thighs.

#### Ice treatment

Inoculated thighs were placed in the coolers (Igloo 48-Qt Island Breeze Cooler; 64.92 × 35.71 × 35.86 cm) containing different ice treatments. The FICE:Meat ratio was maintained at 2:1 w/w with alternate layers of FICE and meat. The coolers were placed in a walk-in refrigerator maintained at 4 °C.

#### Enumeration of *Salmonella*

Samples were analyzed for the survival of *Salmonella* Typhimurium on XLT4 agar supplemented with 35 μL/mL of nalidixic acid at 0, 12, 24, 36 and 48 h of refrigerated storage. At each sampling time, three thighs per treatment were randomly chosen and placed into separate Whirl-Pak^®^ bags (15.24 × 22.86 cm, 710 mL, Whirl-Pak, Nasco, Fort Atkinson, Wisconsin, U.S.A.), 1% PBS (30 mL) was added into each bag and the samples were shaken manually for 1 min. After rinsing, all the thighs were removed from the bags using sterile tongs and returned to the ice cooler. Each thigh that had been sampled was placed in a red plastic mesh to make sure that a new sample was chosen at each sampling point. Serial dilutions were prepared from the rinsate, spread plated (0.1 mL) on duplicate XLT4 agar plates containing nalidixic acid (35 μL/mL) and the plates were incubated at 37 °C for 24 h. Viable colonies showing typical *Salmonella* colony morphology were counted and reported as log CFU/mL of the rinsate. Log differences for each treatment were computed individually as the difference in *Salmonella* counts observed at 0 and 48 h of storage. Experimental design for the study was as follows: 5 treatments × 5 sampling time points × 3 samples/sampling time point/treatment × 5 trials (n = 15 samples/sampling time/treatment × 5 trials). After the end of each trial, coolers were disinfected with 10% bleach solution.

### Effect of FICE on the weight pick-up (%) of thighs during storage

Fresh, boneless, skinless raw poultry thighs were obtained from the same commercial poultry processor. The experimental design for the study was 5 treatments × 5 sampling points × 15 samples/treatment × 3 replications. The meat was stored at 4 °C for approximately 2–3 h until treatments were applied by packing the thighs in coolers with FICE. The thighs were tagged to provide a unique identification number to each thigh and to keep track of their weight throughout the 48 h study. At 0 h sampling time point, the weights of all individual thighs were recorded and then placed (n = 15/treatment/trial) in individual coolers (Igloo 48-Qt Island Breeze Cooler; 64.92 × 35.71 × 35.86 cm) with FICE treatments and stored as stated in the previous section. Same set of 15 thigh meat samples were weighed at 12, 24, 36 and 48 h of storage. At each sampling time, all the thighs were removed from the coolers with different FICE treatments, weighed and placed back into the respective coolers. The difference in the weights of the thighs before and after treatment at each sampling time was calculated and reported as percent weight pick-up [26].

### Effect of FICE on the microbial shelf life and quality of tray-packed thigh meat

Based on the results from *Salmonella* survival study, the STPP 5% and SL-SD 2.5% FICE treatments and control ice were selected for this experiment. Fresh boneless, skinless chicken thighs were obtained from the commercial poultry processor and stored at 4 °C for 13–15 h until further treatment. Thigh meat (n = 180/treatment × 3 trials) was stored in respective FICE and ice treatments in a cooler (Coleman, 52-Quart Xtreme 5-Day Heavy-Duty Cooler; 68.83 × 38.1 × 44.20 cm) (n = 45 thighs/cooler; FICE: Meat: 2:1) for 48 h to simulate the storage period in a processing plant. After 48 h, the thighs were removed from their respective ice treatments using sterile stainless-steel tongs (30.48 cm) and packaged in the Styrofoam trays (23.50 × 18.41 × 6.98 cm; CKF Inc., Hantsport, NS, Canada) (4 pieces/tray) with two absorbent pads (12.7 × 17.78 cm.; tite-dri Industries, Boynton Beach, FL, U.S.A.) wrapped with PVC meat wrapping film (38.1 cm; 60 gauge; Prime Source, St. Louis, MO, U.S.A.) and stored in a walk-in refrigerator (4 °C).

Sampling was conducted on the freshly obtained thighs from the processor to establish a baseline, immediately after 48 h of FICE storage when the thighs were tray-packed (day 0), and every 2-days (day 2, 4, 6 and 8) until the tray-packed samples reached the aerobic plate count limit of 10^7^ CFU/mL of rinsate. Samples were analyzed for microbiological and quality parameters. All the experiments were repeated in three separate trials.

#### Microbiological analyses

Freshly procured raw poultry thigh samples (n = 30 samples × 3 trials) and tray-packed thighs (n = 2 thighs/tray × 5 trays/treatment/sampling day/trial × 3 trials) were analyzed for psychrotrophic plate count (PSY), aerobic plate count (APC), and presumptive lactic acid bacteria (LAB). Individual thigh samples were aseptically placed in a Whirl-Pak^®^ bag and rinsed with 1% phosphate buffered saline (30 mL), serially diluted in PBS and spread plated in duplicate on standard methods agar (PCA, Acumedia manufacturers Inc.,) for APC and PSY, and De Man, Rogosa and Sharpe agar (MRS) (Acumedia Manufacturers Inc.,) for presumptive LAB. PCA plates were incubated at 37 °C for 24 h and at 4 °C for 7–8 days for the estimation of APC and PSY, respectively [27, 28]. The MRS plates were placed in AnaeroPack rectangular jars (28.0 × 21.3 × 11.2 cm.; Mitsubishi Gas Chemical America, Tokyo, Japan) with three anaerobic packs (AnaeroPack^®^ System, Mitsubishi Gas Chemical Company, Inc., New York, NY, U.S.A.) per container and incubated for 48 h at 37 °C. Isolated colonies were counted and reported as log CFU/mL of rinsate.

#### Quality parameters: Color, cook loss, and texture

Freshly procured raw thigh samples (25 samples × 3 trials) and tray-packed thighs (n = 1 thigh/tray × 5 trays/treatment/sampling time/trial × 3 trials) were analyzed for color, cook loss and texture. The objective analysis for color (3 measurements/thigh) was conducted using a Minolta colorimeter (Minolta Corp., model CR-300 / DP301, Ramsey, NJ, U.S.A.) using the CIE: L* (lightness), a* (redness), and b* (yellowness) color spectra [23]. The colorimeter was calibrated with the white calibration plate, with a diffuse illuminant (D65) and 0° viewing geometry and had an 8 mm measurement area. Cook loss is expressed as weight loss after cooking the thigh relative to its initial weight. Briefly, individual thighs were weighed, placed on a raised stainless steel wire rack in a stainless-steel pan (53.02 × 32.54 × 10.16 cm; Vollrath Co., LLC, Sheboygan, WI, U.S.A.), covered with aluminum foil and cooked in a pre-heated (176.6 °C) forced air convection oven (Vulcan HEC5D, Troy, OH, U.S.A.) to an internal temperature of 74 °C [29] measured using a stainless-steel digital thermometer (Taylor 1470FS Digital cooking thermometer and Kitchen Timer, Las Cruces, NM, U.S.A.). After cooking, the thighs were cooled to room temperature (22 ± 2 °C) in the covered pans and then reweighed. Cook loss was calculated using the following formula:

$$\text{Cook}\;\text{loss}\;\left(\%\right)=100\times \frac{\left(Initial\;weight\;of\;thigh-cooked\;weight\;of\;thigh\right)}{\left(Initial\;weight\;of\;thigh\right)}$$

After recording the post-cook weight, the thighs were stored in resealable plastic bags overnight in the walk-in refrigerator at 4 °C for further analysis. The following day, the cooked thighs were brought to room temperature (22 ± 2 °C) for texture analysis. A Warner- Bratzler (WB) knife with guillotine block (TA-7, Stable Micro Systems, Hamilton, MA, U.S.A.) was utilized to shear the samples perpendicular to the muscle fibers. The tenderness of the thighs was evaluated by average peak force using the TA.XTPlus Texture Analyzer (Texture Technologies Corp., Hamilton, MA/Stable Micro Systems, Godalming, Surrey, UK) connected to a computer for obtaining data and analysis via Texture Expert software. The texture analyzer was calibrated using a load cell of 50 kg and at a crosshead speed of 20 mm/sec. From each thigh (*M*. *Iliotibialis lateralis)* two strips of approximately 2.5 × 0.5–0.7 cm were cut (long axis parallel to muscle fibers) using a knife and placed under the ‘V’ slot of WB blade. Average peak force (kg) was measured and utilized as a measurement of thigh tenderness [30]. The entire experiment was conducted in three separate trials.

#### Effect of FICE storage on the pH and temperature of thigh meat

Freshly deboned thighs (n = 45/treatment × 3 trials) were placed in different FICE treatments (STPP 5% and SL-SD 2.5%) and traditional ice (Control ice) in coolers and placed in walk-in refrigerator (4 °C) for 48 h. Thigh samples from each treatment (n = 3 thighs/treatment/sampling time/trial) were analyzed for pH (Hach, Model No. H170G & PHW57-SS, Loveland, CO, U.S.A.) at 0 and 48 h while temperature was recorded using a digital thermometer (Taylor 1442 Critical Care Digital Thermometer with Dual Probes # 6081442, Lancaster, PA, U.S.A.) at 0, 4, 6, 8, 10, 12, 24, 30, 36 and 48 h of storage by inserting the probe in the middle of thigh meat sample. These experiments were repeated in three separate trials.

### Statistical analysis

Data analysis was performed using ANOVA with treatment and sampling time as main effects in the general linear model of SAS (SAS 9.4 Institute, Inc.). Statistical differences between treatments at a particular sampling time and for each treatment over the sampling period for the various parameters were reported as least square means and significance was reported at a level of p ≤ 0.05. For statistical analysis of the microbial data, values of 0 were replaced with the minimum detection limit of 5 CFU/mL.

## Results and discussion

### Effect of FICE on the survival of *Salmonella* Typhimurium inoculated on thigh meat

The initial population of *Salmonella* Typhimurium on the inoculated thigh meat samples was 6.8 to 6.9 log CFU/mL of rinsate (Table 1) which reduced by 0.9 log CFU/mL of rinsate in in the control ice and STPP 2.5% FICE at the end of 48 h refrigerated storage. Comparatively, STPP 5% FICE reduced *Salmonella* by 0.9 logs in 12 h and 1.21 logs in 48 h of storage (p ≤ 0.05). Antimicrobial properties of phosphates can be attributed to (i) ability to sequester divalent metallic cations from the cell wall itself or the nutrient medium making them unavailable for physiological processes; (ii) disruption of proteins involved in cell division; and (iii) causing cell lysis leading to cell death [31, 32]. Furthermore, the level of decline in bacterial population depends on variables such as contact time between the chicken meat and antimicrobials, concentration or the amount of antimicrobials used, method of application, and the temperature of antimicrobial solution [33, 34]. In the current study, the thigh meat samples were in constant contact with the FICE containing polyphosphates and the temperature of FICE made of tripolyphosphate was about -1.55 °C at the end of 48 h of storage (Fig 4). Antimicrobial effect of phosphate is well documented. Chilling the carcasses in 1% and 1.5% Brifisol K^™^ (a commercial blend of sodium acid pyrophosphate and orthophosphoric acid) ice water solution has been found to reduce the incidence of *Salmonella* Typhimurium on inoculated carcasses by 100% and 97.5% respectively, whereas, chilling in only ice water reduced *Salmonella* incidence by 57.5% [35]. Foster and Mead reported a reduction in the survival of *Salmonella* to 0.003–0.24% in the chicken breast muscle injected with 5% polyphosphate (w/v) (Puron 604) stored at -2 °C as compared to the survival of approx. 4% in the non-injected control samples [36].

**Table 1:** Effect of different FICE^1^ treatments and control ice^2^ on the survival of *Salmonella* Typhimurium (log CFU/mL of rinsate) inoculated on thigh meat (n = 75/ trt.) over 48 h of refrigerated storage at 4 °C (mean; SEM: Standard error of means).

Treatments	Storage time (h)					SEM
	0	12	24	36	48	
**Control**^2^	6.90^b^^a^^,^^A^	6.36^a^^,^^B^	6.26^b^^a^^,^^B^	6.10^b^^a^^,^^C^	5.96^a^^,^^D^	0.045
**STPP 2.5%**	6.87^b^^a^^,^^A^	6.03^c^^,^^B^	5.95^c^^,^^B^	5.93^b^^,^^B^	5.93^a^^,^^B^	0.068
**STPP 5%**	6.81^b^^,^^A^	5.84^d^^,^^B^	5.74^d^^,^^B^	5.69^c^^,^^B^	5.69^b^^,^^B^	0.061
**SL-SD 1%**	6.97^a^^,^^A^	6.22^b^^a^^,^^B^	6.27^a^^,^^B^	6.22^a^^,^^B^	5.85^b^^a^^,^^C^	0.055
**SL-SD 2.5%**	6.87^b^^a^^,^^A^	6.17^b^^c^^,^^B^	6.10^b^^c^^,^^C^^B^	5.94^b^^,^^C^^D^	5.85^b^^a^^,^^D^	0.063
**SEM**	0.047	0.055	0.055	0.066	0.069	

^a-e^Means with the different letter within a column indicate the significant differences (p ≤ 0.05) between different treatments at each sampling time point

^A-D^Means with the different letter within the same row indicate the significant differences (p ≤ 0.05) within the treatment at different sampling time point

^1^FICE Treatments: sodium tripolyphosphate (STPP 2.5% and STPP 5%); sodium lactate-sodium diacetate (SL-SD 1% and SL-SD 2.5%)

^2^Control Ice: Regular Ice made of Potable Water

The treatments SL-SD 1% and SL-SD 2.5% exhibited bactericidal activity and reduced *Salmonella* levels on inoculated thighs by approximately 1 log after 48 h of storage (p ≤ 0.05), however there were no statistical differences between the two concentrations of SL-SD and control (p > 0.05). Similar observations were made by Mbandi & Shelef who reported bactericidal effect of SL and SD against *Salmonella* inoculated in sterile comminuted beef [37]. Shelef also stated the antimicrobial activity of the lactates could be attributed to the lowering of water activity and pH [38]. However, in the current study, no differences were observed in the surface pH of the control and SL-SD FICE treated thigh meat (p > 0.05). Another theory stated that the antimicrobial activity of salts of organic acids could be correlated with the acidification of the microbial cells due to the dissociation of undissociated forms of weak lipophilic acids into the microbial cells [39]. However, there is not much published literature on the antimicrobial activities of a combination of sodium lactate and sodium diacetate against *Salmonella* in raw poultry meat.

### Effect of FICE on the weight pick-up (%) of thighs during storage

The control and SL-SD 1% exhibited lowest weight pick-up (%) compared to SL-SD 2.5% and STPP 5% with a weight pick-up ranging from 7.43 to 7.65% (p < 0.05) (Table 2). Increased yield could be attributed to the ability of organic acid salts to cause swelling in the myofibrillar proteins causing higher water accumulation in the muscle [30]. However, limited documentation explaining the exact mechanism of higher yield due to lactates and diacetates is unknown. The improved water holding capacity of STPP FICE can be attributed to the elevated pH, increased ionic strength, protein-ion interactions and hydration, allowing more water to penetrate into muscle structure [40, 41, 42].

**Table 2:** Weight pickup (%) of raw thighs stored in FICE^1^ treatments and control ice^2^ over 48 h of refrigerated storage at 4 °C (mean; SEM: Standard error of means).

Treatment	Weight Pick-Up (%)				SEM
	0 to 12 h	0 to 24 h	0 to 36 h	0 to 48 h	
**Control**^2^	-0.54^e^^,^^C^	-0.28^d^^,^^C^	0.94^d^^,^^B^	2.47^c^^,^^A^	0.199
**STPP 2.5%**	1.06^c^^,^^D^	1.82^c^^,^^C^	3.07^c^^,^^B^	3.99^b^^,^^A^	0.172
**STPP 5%**	1.48^b^^,^^D^	2.96^b^^,^^C^	5.46^b^^,^^B^	7.43^a^^,^^A^	0.204
**SL-SD 1%**	0.59^d^^,^^C^	1.44^c^^,^^A^	0.96^d^^,^^B^^C^	1.09^d^^,^^B^^A^	0.169
**SL-SD 2.5%**	2.79^a^^,^^C^	4.48^a^^,^^B^	6.88^a^^,^^A^	7.65^a^^,^^A^	0.283
**SEM**	0.147	0.175	0.226	0.270	

^a-e^Means with the different letter within a column indicate the significant differences (p ≤ 0.05) between different treatments at each sampling time point

^A-D^Means with the different letter within the same row indicate the significant differences (p ≤ 0.05) within the treatment at different sampling time point

^1^FICE Treatments: sodium tripolyphosphate (STPP 2.5% and STPP 5%); sodium lactate-sodium diacetate (SL-SD 1% and SL-SD 2.5%)

^2^Control Ice: Regular Ice made of Potable Water

### Effect of FICE on the microbial shelf life and quality of tray-packed thigh meat

#### Microbiological analyses (PSY, APC, and LAB)

The effect of refrigerated storage of thigh meat in STPP 5%, SL-SD 2.5% and control ice treatment for 48 h, on microbiological shelf life (PSY, APC, and LAB) of tray-packed product was monitored for 8 d. Thigh meat samples were regarded as unacceptable when APC and PSY levels reached 10^7^ CFU/mL of the rinsate.

**Fig 1:**
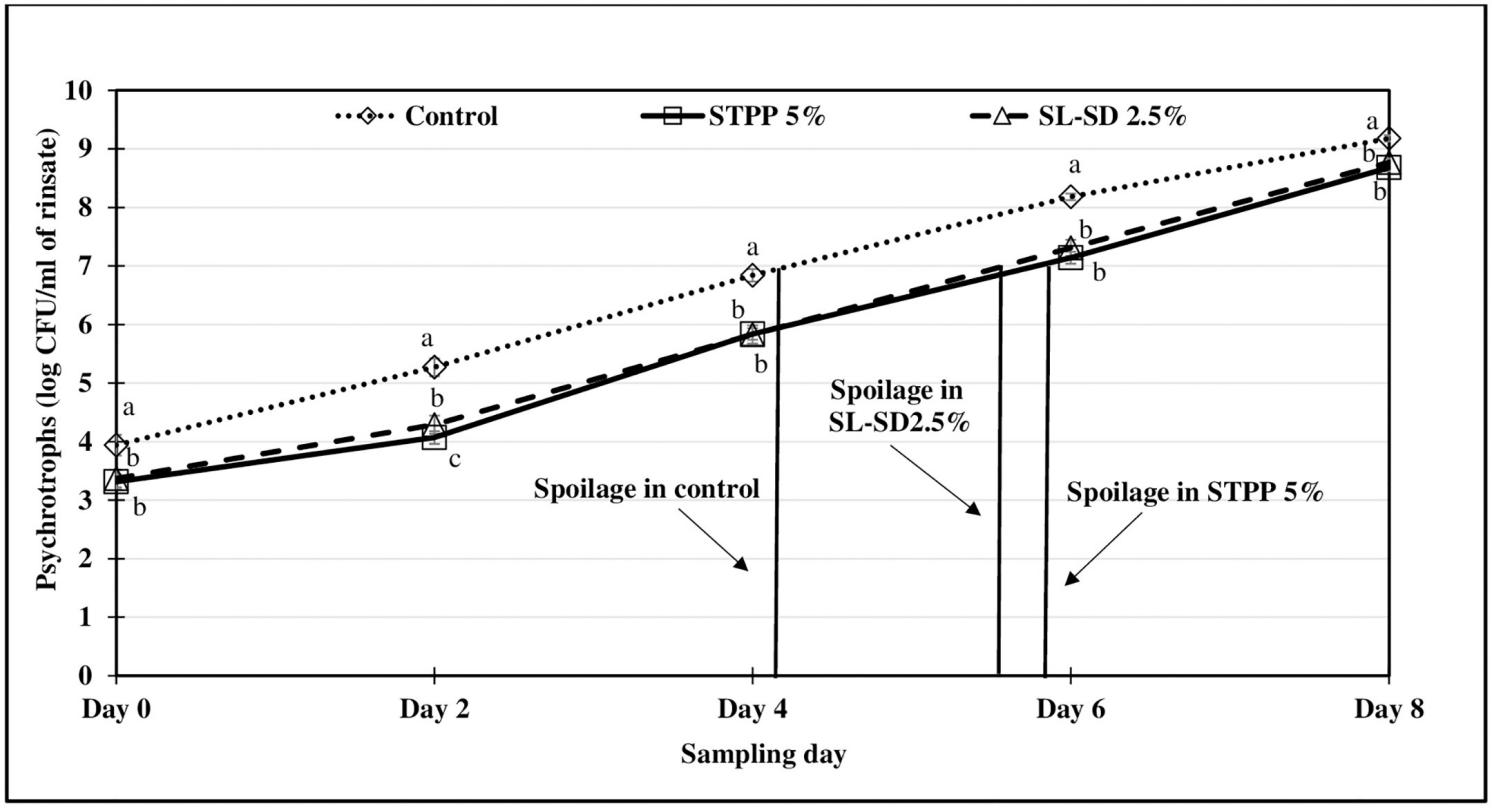
Psychrotroph counts (log CFU/mL of rinsate) of tray-packed, FICE-treated thighs during refrigerated storage (4 °C) for 8 days (Psychrotrophic count of freshly procured thigh meat was 3.49 ± 0.62 log CFU/mL of rinsate). ^a-c^Means with the different letters indicate the significant differences (p ≤ 0.05) between different treatments.

**Fig 2:**
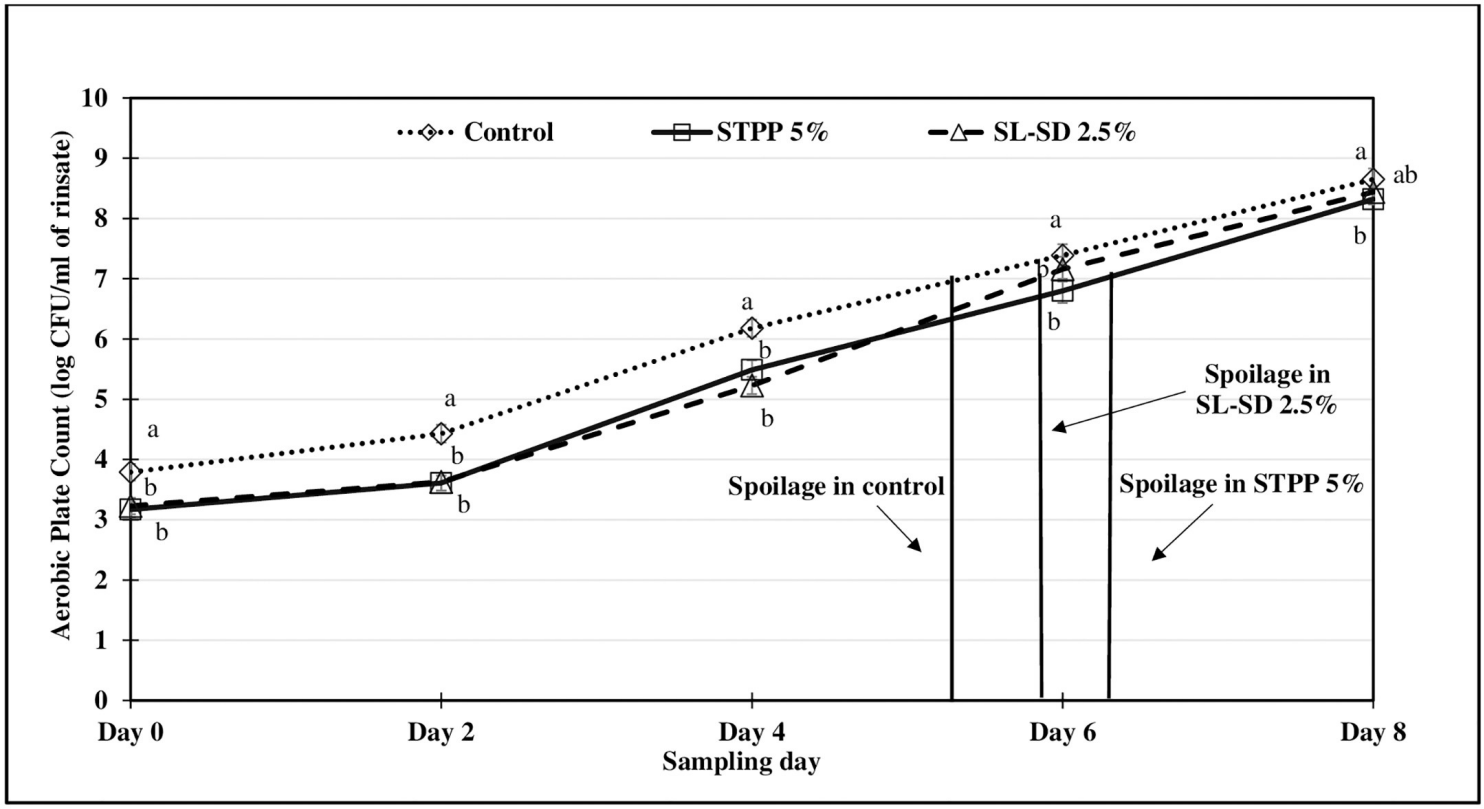
Aerobic plate count (log CFU/mL of rinsate) of tray-packed, FICE-treated thighs during refrigerated storage (4 °C) for 8 days (Aerobic plate count of freshly procured thigh meat was 3.47 ± 0.531 log CFU/mL of rinsate). ^a-c^Means with the different letter indicate the significant differences (p ≤ 0.05) between different treatments.

**Fig 3:**
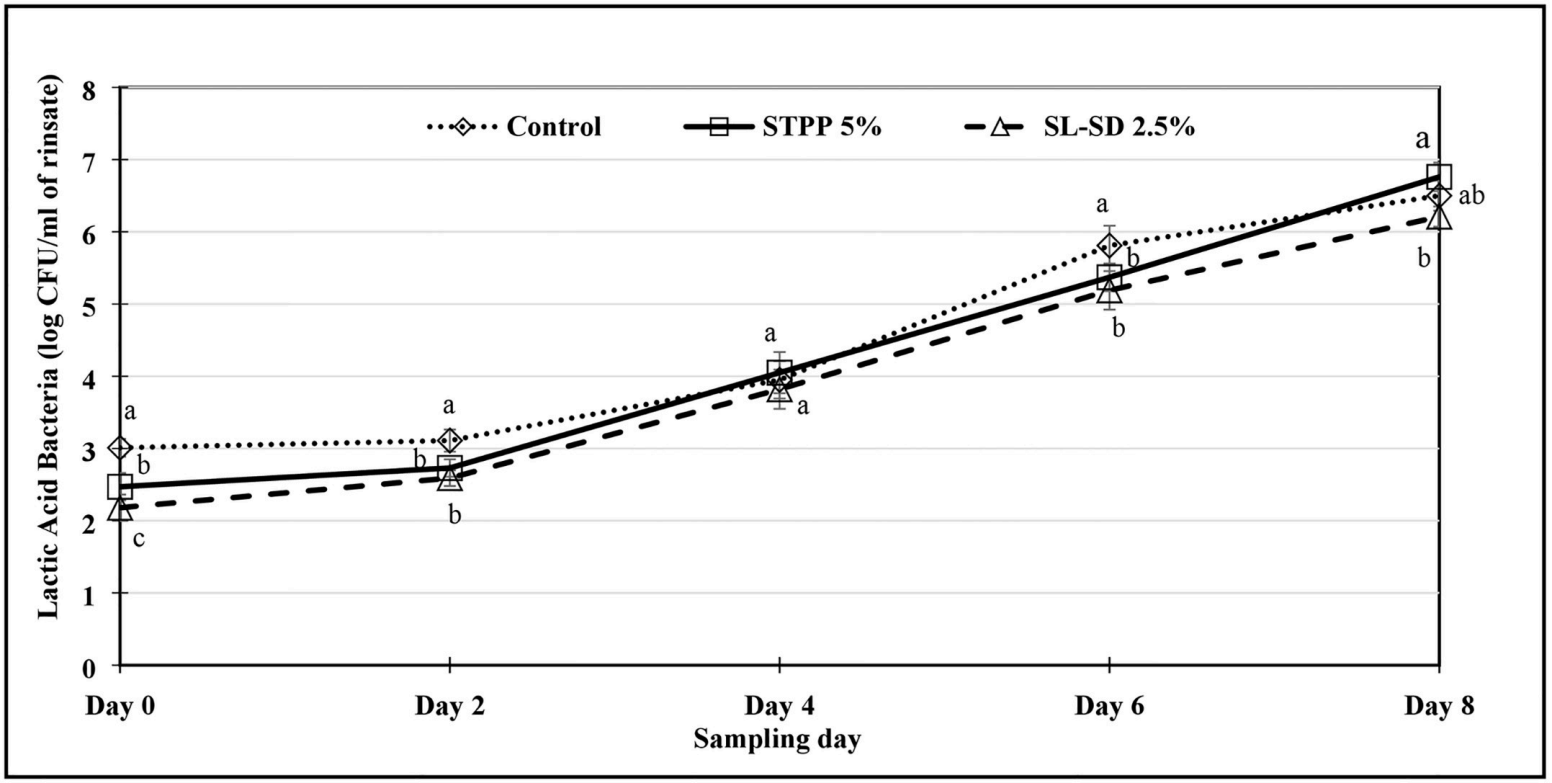
Lactic acid bacteria count (log CFU/ml of rinsate) of tray-packed, FICE-treated thighs during refrigerated storage (4 °C) for 8 days (Lactic acid bacteria count of freshly procured thigh meat was 2.63 ± 0.697 log CFU/mL of rinsate). ^a-c^Means with the different letter indicate the significant differences (p ≤ 0.05) between different treatments on the same sampling day.

**Fig 4:**
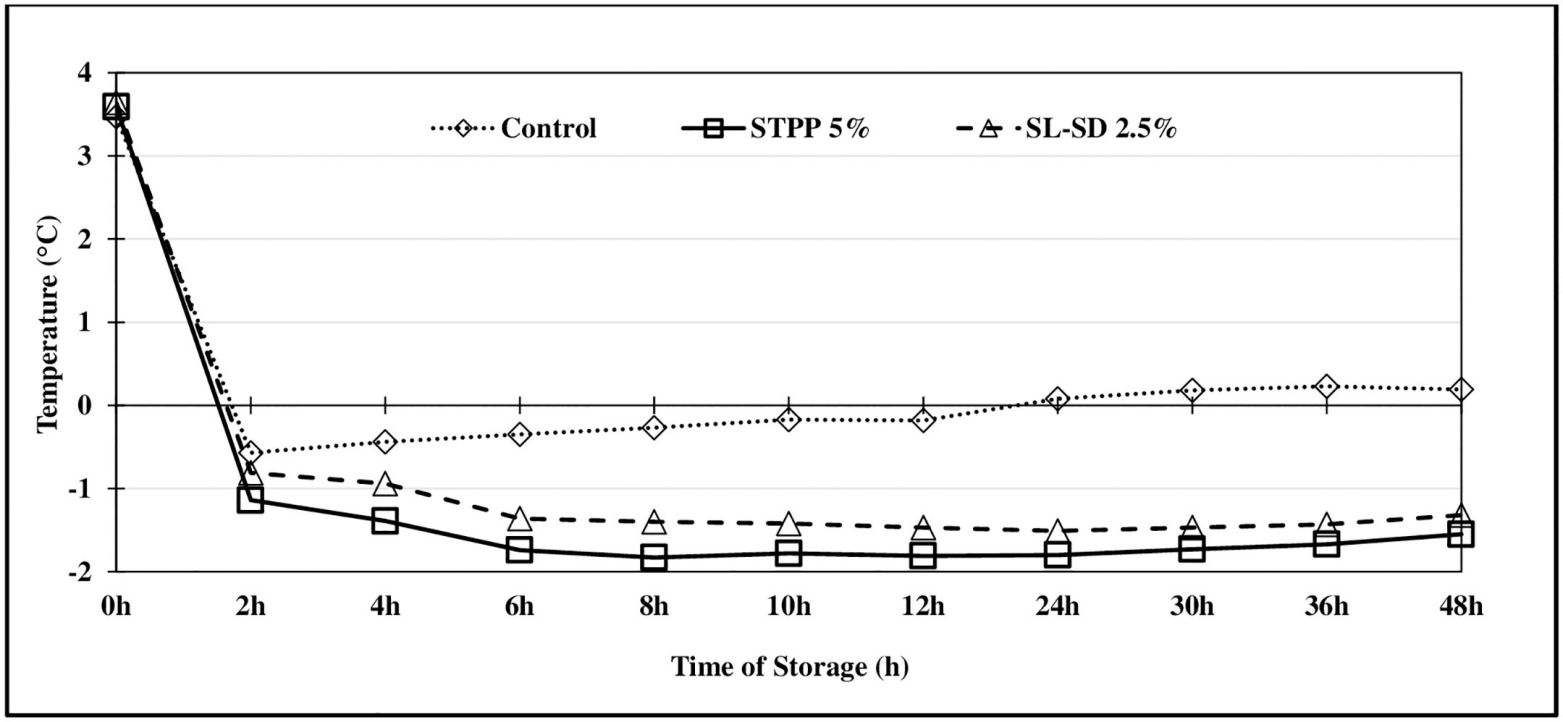
Temperature change recorded in the thighs stored in control ice and different FICE treatments over 48 h of refrigerated storage at 4 °C.

Aerobic plate counts (APC) are commonly used as an indicator of microbiological spoilage in the food industry while psychrotrophic specific spoilage microorganisms (SSO) such as *Pseudomonas fluorescence* and *Shewanella putrefaciens* cause spoilage odors and flavors rendering the food unacceptable for consumption [43]. Since raw poultry is stored under refrigeration, it would be important to determine the PSY as a representation of the SSOs. Fresh thigh meat had an PSY and APC of approx. 3–4 log CFU/mL of rinsate while the presumptive LAB was approx. 2–3 log CFU/mL of rinsate. As expected, FICE treated samples, especially STPP 5% had lower APC, PSY and LAB counts compared to the control samples (Figs 1, 2 and 3) at the beginning of the tray-pack study. The control samples reached the PSY (Fig 1) and APC (Fig 2) spoilage limit 1–2 days sooner than the FICE treated samples attributed to potential antimicrobial effect of residual STPP and SL-SD in meat. LAB counts increased exponentially after day 2 with significant differences among the treatments, however the differences in the counts were minimum (Fig 3). Similar shelf life enhancement effects of polyphosphates were reported by Vareltzis et al. in chicken carcasses dipped in 5% STPP (w/v) solution for 10 min [44]. This indicates that the antimicrobial efficacy of polyphosphates greatly depends on the method of polyphosphate application and contact time [18]. Smaoui et al. reported a 3 d extension in the shelf life of chicken thighs marinated with 3% sodium lactate [45]. Williams et al. also reported lower levels of APC for catfish fillets when a higher concentration of SL was used [20]. In addition to the antimicrobial effect of STPP and SL-SD, FICE-stored thigh meat had lower temperature (< -1°C) compared to the control ice (-0.5 to 0°C) during the 48-h storage period (Fig 4) which could have further led to lower microbial growth in FICE-treated samples.

#### Quality analyses

*Color*. The food ingredients used in the FICE may impact color of the meat which can ultimately influence the consumer selection of the product [46]. The L* values in the control and SL-SD samples were higher than STPP 5% treatment until d 2 (p ≤ 0.05), while on d 4, 6 and 8, no differences were observed (p > 0.05; Table 3). There were no differences (p > 0.05) in the a* values of control and SL-SD samples throughout the tray-pack storage for 8 d [47, 48], indicating no adverse impact on the redness of the samples. The b* values for the control and SL-SD samples were fairly similar throughout the tray-packed storage (p > 0.05) but higher (indicating yellower samples) than STPP 5% samples until d 8 (p ≤ 0.05). Thus, the tray-pack storage of different FICE treated samples denoted a similar L* and b* value (p > 0.05), whereas, a comparatively lower a* (less red) value was observed for STPP 5% treatment by the end of the study.

**Table 3:** Effect of refrigerated storage (4 °C) on the color (L*, a*, and b*) of tray-packed, FICE^1^-treated thigh meat (n = 75/trt.) (mean; SEM: Standard error of means).

Treatment	Parameter	Fresh, raw thigh meat (untreated)	Storage time (day)					SEM
			0	2	4	6	8	
**Control**^2^	L*	51.43	59.23^a^^,^^A^	57.07^a^^,^^B^	54.12^b^^a^^,^^C^	53.61^a^^,^^C^	52.97^a^^,^^C^	0.709
**STPP 5%**			52.75^c^^,^^B^^A^	51.79^c^^,^^B^	52.87^b^^,^^B^^A^	53.50^a^^,^^B^^A^	53.81^a^^,^^A^	0.703
**SL-SD 2.5%**			56.67^b^^,^^A^	55.52^b^^,^^B^^A^	55.51^a^^,^^B^^A^	53.81^a^^,^^B^	53.83^a^^,^^B^	0.671
**SEM**			0.861	0.523	0.861	0.559	0.585	
**Control**^2^	a*	4.29	2.70^a^^,^^A^	2.57^a^^,^^A^	2.77^a^^,^^A^	2.22^b^^a^^,^^A^	2.96^a^^,^^A^	0.296
**STPP 5%**			2.67^a^^,^^A^	2.45^a^^,^^B^^A^	1.96^a^^,^^B^^C^	1.65^b^^,^^C^	1.41^b^^,^^C^	0.206
**SL-SD 2.5%**			2.24^a^^,^^B^	2.51^a^^,^^B^^A^	2.53^a^^,^^B^^A^	2.61^a^^,^^B^^A^	3.01^a^^,^^A^	0.259
**SEM**			0.274	0.235	0.333	0.212	0.205	
**Control**^2^	b*	3.52	6.92^a^^,^^A^	7.28^a^^,^^A^	5.15^b^^,^^B^	6.73^a^^,^^B^^A^	5.12^a^^,^^B^	0.573
**STPP 5%**			4.96^b^^,^^A^	3.90^b^^,^^A^	4.88^b^^,^^A^	4.57^b^^,^^A^	5.63^a^^,^^A^	0.636
**SL-SD 2.5%**			6.28^a^^,^^A^	6.23^a^^,^^A^	7.29^a^^,^^A^	6.24^a^^,^^A^	6.32^a^^,^^A^	0.578
**SEM**			0.676	0.592	0.612	0.479	0.604	

^a-c^Means with the different superscript within the same column and parameter indicate the significant differences (p ≤ 0.05) between different treatments on the same sampling day

^A-C^Means with the different superscript within the same row indicate the significant differences (p ≤ 0.05) within the treatment on different sampling days

^1^FICE Treatments: sodium tripolyphosphate (STPP 5%); sodium lactate-sodium diacetate (SL-SD 2.5%)

^2^Control Ice: Regular Ice made of Potable Water

*Cook loss and texture*. The cook loss of fresh meat was 29.15% which increased after a 48 h storage in control ice and SL-SD 2.5% FICE (Table 4), while it was lower in STPP 5% FICE treatment (26.53%; p ≤ 0.05) indicating the impact of FICE on the quality of raw poultry meat during storage. Cooking losses for control and SL-SD 2.5% samples were comparable throughout the study (p > 0.05) except for day 8 when the control exhibited the highest cook loss. By the end of study (d 8) STPP 5% exhibited the lowest cook loss (14.86%; p ≤ 0.05) in the tray-packed meat. Sodium tripolyphosphate results in increased water retention, reduced cooking losses and indirectly impacting texture of chicken meat [15, 16, 40, 41, 47, 49, 50]. While some texture differences in this study were significantly different (Table 4), the differences were small and would likely go unnoticed by a consumer.

**Table 4:** Effect of refrigerated storage (4 °C) on the cook loss (%) and peak force (kg) values of tray-packed, FICE^1^-treated thigh meat (n = 75/trt.) (mean; SEM: Standard error of means).

Treatment	Parameter	Fresh, raw thigh meat (untreated)	Storage time (day)					SEM
			0	2	4	6	8	
**Control**^2^	**Cook loss (%)**	29.15	33.02^a^^,^^A^	32.10^a^^,^^A^	27.12^a^^,^^B^	27.96^a^^,^^B^	27.09^a^^,^^B^	0.765
**STPP 5%**			26.53^b^^,^^A^	23.01^b^^,^^B^	18.32^b^^,^^C^	19.06^b^^,^^C^	14.86^c^^,^^D^	0.650
**SL-SD 2.5%**			35.89^a^^,^^A^	31.44^a^^,^^B^	27.19^a^^,^^C^	27.51^a^^,^^C^	21.94^b^^,^^D^	1.136
**SEM**			1.006	1.272	0.617	0.719	0.548	
**Control**^2^	**Peak force (kg)**	1.5	1.13^b^^a^^,^^A^	1.08^a^^,^^B^^A^	0.91^a^^,^^B^^C^	0.82^b^^,^^C^	0.75^a^^,^^C^	0.070
**STPP 5%**			0.97^b^^,^^A^	0.91^a^^,^^B^^A^	0.91^a^^,^^B^^A^	1.03^b^^a^^,^^A^	0.77^a^^,^^B^	0.064
**SL-SD 2.5%**			1.24^a^^,^^A^	1.10^a^^,^^A^	0.78^a^^,^^C^^B^	0.89^a^^,^^B^	0.69^a^^,^^C^	0.058
**SEM**			0.078	0.071	0.059	0.069	0.034	

^a-c^Means with the different superscript within the column and parameter indicate the significant differences (p ≤ 0.05) between different treatments on the same sampling day

^A-D^Means with the different superscript within the same row indicate the significant differences (p ≤ 0.05) within the treatment on different sampling days

^1^FICE Treatments: sodium tripolyphosphate (STPP 5%); Sodium lactate-sodium diacetate (SL-SD 2.5%)

^2^Control Ice: Regular Ice made of Potable Water

#### Effect of FICE storage on the pH and temperature of thigh meat

The pH of fresh, raw poultry thigh meat was 6.55. After 48 h of treatment, the pH of control (6.69) and SL-SD 2.5% (6.85) treated thigh meat samples was not significantly different (p > 0.05). On the contrary, treatment of thigh meat with 5% STPP FICE for 48 h significantly increased (p ≤ 0.05) the pH to 7.84. The increase in the pH of thighs stored in STPP can be attributed to the alkaline nature of STPP [50].

Temperature of thighs stored in in STPP 5% FICE showing the lowest temperature decrease within 2 hours (-1.14 °C; p ≤ 0.05) (Fig 4). On the other hand, compared to control, the FICE treatments exhibited lower temperatures (p ≤ 0.05) of -0.94 to -1.55 °C at the end of 48 h of storage. The different FICE ingredients further resulted in a depression in the freezing point of the ice, enhancing the cooling capacity of the FICE and thereby increasing the rate of heat transfer and lowering the temperature of meat.

## Conclusion

Functional ice made with STPP 5% was effective against *Salmonella* Typhimurium in the first 12 h of storage but exhibited comparative reductions after 48 h storage. In addition, STPP 5% had a significant impact on improving yield, quality, and shelf life of raw thigh meat. SL-SD 2.5% was the second most effective FICE treatment but was not as effective as the STPP treatment. FICE could be applied during storage and transportation of raw poultry to not only provide an additional hurdle to ensure food safety and shelf life extension but also improve the quality characteristics of poultry meat.

## Supporting information

S1 Data (ZIP)

## References

[1] Center for Disease Control and Prevention 2018. Outbreak of Salmonella infections linked to chicken. https://www.cdc.gov/salmonella/chicken-08-18/index.html

[2] Report MW. Surveillance for foodborne disease outbreaks- United States, 2009–2015. MMWR Surveill Summ. 2018; 67(10): 1 10.15585/mmwr.ss6710a1 PMC6061962 30048426

[3] Rouger A, Tresse O, Zagorec M. Bacterial contaminants of poultry meat: sources, species, and dynamics. Microorganisms. 2017; 5(3): 50. 10.3390/microorganisms5030050 PMC5620641 28841156

[4] Dominguez SA, Schaffner DW. Survival of *Salmonella* in processed chicken products during frozen storage. J Food Prot. 2009; 72(10): 2088–2092. 10.4315/0362-028x-72.10.2088 19833031

[5] Lee S, Pintar K, Cook A, Pollari F. quantitative effect of refrigerated storage time on the enumeration of *Campylobacter*, *Listeria*, and *Salmonella* on Artificially inoculated raw chicken meat. J Food Prot. 2007; 70(3): 739–743. 10.4315/0362-028x-70.3.739 17388068

[6] Palumbo SA. Is refrigeration enough to restrain foodborne pathogens? J Food Prot. 1986; 49(12):1003–1009. 10.4315/0362-028X-49.12.1003 30965458

[7] Lambert AD, Smith JP, Dodds KL. Shelf life extension and microbiological safety of fresh meat- A review. Food Microbiol. 1991; (8): 267–297.

[8] Graham J, Johnston WA, Nicholson FJ. Ice in Fisheries (No. 331). Food and Agriculture Org. 1992. Retrieved from http://innri.unuftp.is/short_courses/safety_qm_srilanka06/Additional%20material/Ice%20in%20fisheries_FAO.pdf

[9] Fratamico PM, Juneja V, Annous BA, Rasanayagam V, Sundar M, Braithwaite D, et al Application of Ozonated Dry Ice (ALIGAL^™^ Blue Ice) for packaging and transport in the food industry. J Food Sci. 2012; 77(5): 285–291. 10.1111/j.1750-3841.2012.02682.x 23163945

[10] Jeyasekaran G, Ganesan P, Shakila RJ, Maheswari K, Sukumar D. Dry ice as a novel chilling medium along with water ice for short-term preservation of fish Emperor breams, lethrinus (*Lethrinus miniatus*). Innov Food Sci Emerg Technol. 2004; 5(4): 485–493.

[11] Bragg RJ. Shipment of perishable products and dry ice usage. Centre for Profitable Agriculture, UT Extensions, CPA Info # 81. 2003: 1–5.

[12] Harvey MS, Howarth JN. Antimicrobial Ice compositions, methods of preparation and methods of use. United States Patent Application Publication US 2007/0184155 A1. 2007.

[13] Pechacek N, Osorio M, Caudill J, Peterson B. Evaluation of the toxicity data for peracetic acid in deriving occupational exposure limits: A minireview. Toxicol Letters. 2015; 233: 45–57. 10.1016/j.toxlet.2014.12.014 25542141

[14] United States Department of Agriculture, Food Safety and Inspection Service. Food Additives for use in meat and poultry products: Sodium diacetate, sodium acetate, sodium lactate and potassium lactate. Direct final rule. Federal Register. 2000; 65: 3121–3123.

[15] Alvarado C, McKee SR. Marination to improve functional properties and safety of poultry meat. J Appl Poult Res. 2007; 16(1): 113–120.

[16] Smith DP, Young LL. Marination pressure and phosphate effects on broiler breast fillet yield, tenderness, and color. Poult Sci. 2007; 86(12): 2666–2670. 10.3382/ps.2007-00144 18029814

[17] Lee RM, Hartman PA, Stahr HM, Olson DG, Williams FD. Antibacterial mechanism of long-chain polyphosphates in *Staphylococcus aureus*. J Food Prot. 1994b; 57(4): 289–294. 31113131 10.4315/0362-028X-57.4.289

[18] Elliott RP, Straka RP, Garibaldi JA. Polyphosphate inhibition of growth of *Pseudomonads* from poultry meat. Appl Microbiol. 1964; 12(6): 517–522. 14239583 10.1128/am.12.6.517-522.1964 PMC1058171

[19] Zeitoun AAM, Debevere JM. Decontamination with lactic acid/sodium lactate buffer in combination with modified atmosphere packaging effects on the shelf life of fresh poultry. Int J Food Microbiol. 1992; 16(2): 89–98. 10.1016/0168-1605(92)90001-j 1445762

[20] Williams SK, Rodrick GE, West RL. Sodium lactate affects shelf life and consumer acceptance of fresh catfish (*Icfalurus nebulosus*, *marmoratus*) fillets under simulated retail conditions. J Food Sci. 1995; 60(3): 636–639.

[21] Williams SK, Phillips K. Sodium lactate affects sensory and objective characteristics of tray- packed broiler chicken breast meat. Poult Sci. 1998; 77(5): 765–769. 10.1093/ps/77.5.765 9603367

[22] Papadopoulos LS, Miller R.K., Acuff GR, Vanderzant C, Cross HR. Effect of sodium lactate on sensory, microbial, chemical and physical attributes of cooked roast beef during storage. J Food Sci. 1991; 56(2): 341–347.

[23] Baptista RC, Horita CN, Sant’Ana AS. Natural products with preservative properties for enhancing the microbiological safety and extending the shelf-life of seafood: A review. Food Res Int. 2020; 127: 108762 10.1016/j.foodres.2019.108762 31882098

[24] Bauermeister LJ, Bowers JW, Townsend JC, McKee SR. The microbial and quality properties of poultry carcasses treated with peracetic acid as an antimicrobial treatment. Poult. Sci. 2008; 87(11): 2390–2398. 10.3382/ps.2008-00087 18931192

[25] Nagel GM, Bauermeister LJ, Bratcher CL, Singh M, McKee SR. *Salmonella* and *Campylobacter* reduction and quality characteristics of poultry carcasses treated with various antimicrobials in a post-chill immersion tank. Int J Food Microbiol. 2013; 165(3): 281–286. 10.1016/j.ijfoodmicro.2013.05.016 23800739

[26] Kin S, Schilling MW, Smith BS, Silva JL, Kim T, Pham AJ, et al Potassium acetate and potassium lactate enhance the microbiological and physical properties of marinated catfish fillets. J Food Sci. 2011; 76(4): 242–250. 10.1111/j.1750-3841.2011.02122.x 22417369

[27] Shekarforoush SS, Basiri S, Ebrahimnejad H, Hosseinzadeh S. Effect of chitosan on spoilage bacteria, *Escherichia coli* and *Listeria monocytogenes* in cured chicken meat. Int J Biol Macromol. 2015; 76: 303–309. 10.1016/j.ijbiomac.2015.02.033 25735728

[28] Morey A, Bowers JWJ, Bauermeister LJ, Singh M, Huang TS, Mckee SR. Effect of salts of organic acids on *Listeria monocytogenes*, shelf life, meat quality, and consumer acceptability of beef frankfurters. J Food Sci. 2014; 79(1): 54–60. 10.1111/1750-3841.12220 24460770

[29] Saha A, Perumalla AVS, Lee Y, Meullenet JF, Owens CM. Tenderness, moistness, and flavor of pre- and postrigor marinated broiler breast fillets evaluated by consumer sensory panel. Poult Sci. 2009; 88(6): 1250–1256. 10.3382/ps.2008-00236 19439637

[30] Lee N, Sharma V, Brown N, Mohan A. Functional properties of bicarbonates and lactic acid on chicken breast retail display properties and cooked meat quality. Poult Sci. 2015; 94(2): 302–310. 10.3382/ps/peu063 25589078

[31] Lee RM, Hartman PA, Olson DG, Williams FD. Bactericidal and bacteriolytic effects of selected food-grade phosphates, using *Staphylococcus aureus* as a model system. J Food Prot. 1994a; 283(4): 276–283. 10.4315/0362-028X-57.4.276 31113135

[32] Buňková L, Pleva P, Buňka F, Valášek P, Kráčmar S. Antibacterial effects of commercially available phosphates on selected microorganisms. Acta Univ Agric Silvic mendelianae Brun. 2008; 56(5): 19–24.

[33] Hill, J. C., Ivey, F. J., 1988. “Control of Salmonella on poultry carcasses”. U.S. Patent No. 4,770,884.

[34] Dickson JS, Anderson ME. Microbiological decontamination of food animal carcasses by washing and sanitizing systems: A review. J Food Prot. 1992; 55(2): 133–140. 10.4315/0362-028X-55.2.133 31071772

[35] Rathgeber BM, Waldroupl AMYL. Antibacterial activity of a sodium acid pyrophosphate product in chiller water against selected bacteria on broiler carcasses. J Food Prot. 1995; 58(5): 530–534. 10.4315/0362-028X-58.5.530 31137276

[36] Foster RD, Mead GC. Effect of temperature and added polyphosphate on the survival of salmonellae in poultry meat during cold storage. J Appl Bacteriol. 1976; 41(3): 505–510. 10.1111/j.1365-2672.1976.tb00663.x 1035214

[37] Mbandi E, Shelef LA. Enhanced inhibition of *Listeria monocytogenes* and *Salmonella enteritidis* in meat by combinations of sodium lactate and diacetate. J Food Prot. 2001; 64(5): 640–644. 10.4315/0362-028x-64.5.640 11347993

[38] Shelef LA. Antimicrobial effects of lactates: A review. J Food Prot. 1994; 57(5): 445–450. 10.4315/0362-028X-57.5.445 31121747

[39] Salmond CV, Kroll RG, Booth IA. The effect of food preservatives on pH homeostasis in *Escherichia coli*. J Gen Microbiol. 1984; 130(11): 2845–2850. 10.1099/00221287-130-11-2845 6396375

[40] Young LL, Lyon CE. Effect of postchill aging and sodium tripolyphosphate on moisture binding properties, color, and Warner-Bratzler Shear Values of chicken breast meat. Poult Sci. 1997; 76(11): 1587–1590. 10.1093/ps/76.11.1587 9355155

[41] Wynveen EJ, Bowker BC, Grant AL, Lamkey JW, Fennewald KJ, Henson L, et al Pork quality is affected by early postmortem phosphate and bicarbonate injection. J Food Sci. 2001; 66(6): 886–891.

[42] Froning GW. Effect of polyphosphates on binding properties of chicken meat. Poult Sci. 1965; 44:1004–1007. 14343993

[43] Morey A, Himelbloom BH. Bacterial diversity and changes towards spoilage micro flora of iced Alaska pink salmon. J Nutr Heal Food Eng. 2014; 1(1): 25–29.

[44] Vareltzis K, Soultos N, Koidis P, Ambrosiadis J, Genigeorgis C. Antimicrobial effects of sodium tripolyphosphate against bacteria attached to the surface of chicken carcasses. LWT—Food Sci Technol. 1997; 30: 665–669.

[45] Smaoui S, Hlima HB, Salah RB, Ghorbel R. Effects of sodium lactate and lactic acid on chemical, microbiological and sensory characteristics of marinated chicken. African J Biotechnol. 2011; 10(54): 11317–11326.

[46] Fletcher DL. Poultry meat quality. World Poult. Sci Journal. 2002; 58: 131–145.

[47] Carroll CD, Alvarado CZ, Brashears MM, Thompson LD, Boyce J. Marination of turkey breast fillets to control the growth of *Listeria monocytogenes* and improve meat quality in deli loaves. Poult Sci. 2007; 86(1): 150–155. 10.1093/ps/86.1.150 17179430

[48] Shafit HM, Williams SK. Sodium diacetate and sodium lactate affect microbiology and sensory and objective characteristics of a restructured turkey breast product formulated with a fibrin cold-set binding system. Poult Sci. 2010; 89(3): 594–602. 10.3382/ps.2009-00412 20181879

[49] Young LL, Lyon CE Searcy GK, Wilson RL. Influence of sodium tripolyphosphate and sodium chloride on moisture-retention and textural characteristics of chicken breast meat patties. J Food Sci. 1987; 52(3): 571–574.

[50] Cheng Q, Sun D-W. Factors affecting the water holding capacity of red meat products: A review of recent research advances. Crit Rev Food Sci Nutr. 2008; 48(2): 137–159. 10.1080/10408390601177647 18274969

